# Atypical, non-standard functions of the microtubule associated Tau protein

**DOI:** 10.1186/s40478-017-0489-6

**Published:** 2017-11-29

**Authors:** Ioannis Sotiropoulos, Marie-Christine Galas, Joana M. Silva, Efthimios Skoulakis, Susanne Wegmann, Mahmoud Bukar Maina, David Blum, Carmen Laura Sayas, Eva-Maria Mandelkow, Eckhard Mandelkow, Maria Grazia Spillantini, Nuno Sousa, Jesus Avila, Miguel Medina, Amrit Mudher, Luc Buee

**Affiliations:** 1 Life and Health Sciences Research Institute (ICVS), Medical School, University of Minho, Braga, Portugal; 2 ICVS/3B’s - PT Government Associate Laboratory, Guimarães, Braga, Portugal; 3 Univ. Lille, Inserm, CHU Lille, UMR-S 1172 – JPArc, 59000 Lille, France; 4 Division of Neuroscience, Biomedical Sciences Research Centre “Alexander Fleming”, 16672 Vari, Greece; 5 Alzheimer’s Disease Research Laboratory, MassGeneral Institute for Neurodegenerative Disease, Massachusetts General Hospital, Harvard Medical School, Charlestown, MA 02129 USA; 6 School of Life Sciences, University of Sussex, Falmer, Brighton, East Sussex, BN1 9QG UK; 7 Centre for Biomedical Research of the Canary Islands (CIBICAN), Institute for Biomedical Technologies (ITB), Universidad de La Laguna (ULL), Tenerife, Spain; 8 DZNE, German Center for Neurodegenerative Diseases, Bonn, Germany; CAESAR Research Institute, Bonn, Germany; Max-Planck-Institute for Metabolism Research, Köln, Germany; 9 Department of Clinical Neurosciences, Clifford Allbutt Building, Cambridge, AH UK; 10 Centro de Investigación Biomédica en Red de Enfermedades Neurodegenerativas (CIBERNED), Valderrebollo 5, 28041 Madrid, Spain; 11 Centro de Biología Molecular “Severo Ochoa” CSIC-UAM, Universidad Autónoma de Madrid, C/ Nicolás Cabrera 1, 28049 Madrid, Spain; 12 CIBERNED, Network Center for Biomedical Research in Neurodegenerative Diseases, Madrid, Spain; 13 CIEN Foundation, Queen Sofia Foundation Alzheimer Center, Madrid, Spain; 14 Faculty of Natural and Environmental Sciences, University of Southampton Highfield Campus, Center for Biological Sciences, Southampton, UK

**Keywords:** Tau, Alzheimer’s disease, Neuronal function, Pathology, Nucleus, Dendrites, Synapse, Subcellular localization, Tau isoform

## Abstract

Since the discovery of the microtubule-associated protein Tau (MAPT) over 40 years ago, most studies have focused on Tau’s role in microtubule stability and regulation, as well as on the neuropathological consequences of Tau hyperphosphorylation and aggregation in Alzheimer’s disease (AD) brains. In recent years, however, research efforts identified new interaction partners and different sub-cellular localizations for Tau suggesting additional roles beyond its standard function as microtubule regulating protein. Moreover, despite the increasing research focus on AD over the last decades, Tau was only recently considered as a promising therapeutic target for the treatment and prevention of AD as well as for neurological pathologies beyond AD e.g. epilepsy, excitotoxicity, and environmental stress. This review will focus on atypical, non-standard roles of Tau on neuronal function and dysfunction in AD and other neurological pathologies providing novel insights about neuroplastic and neuropathological implications of Tau in both the central and the peripheral nervous system.

## Introduction

Considering the increasing interest of diverse research fields on the role of Tau in brain function and pathology in and beyond Alzheimer’s disease (AD) and the recent focus on Tau-based therapeutic strategies, the 1st EuroTau Meeting was organized in Lille, France on April 27 and 28 April 2017. The meeting attracted many clinical and basic Tau researchers throughout Europe providing a unique forum to discuss and exchange ideas and hypotheses. The meeting facilitated the integration of the diverse findings implicating Tau in neuronal physiology and pathology. During the conference, a round table discussion was held to discuss the emerging various atypical, non-standard functions of Tau protein in the sense of divergence from its cytoskeletal association and beyond AD as it is summarized in this review report.

## Atypical/non-standard functions of Tau

### Tau protein and brain pathology – From past to present

**Fig. 1:**
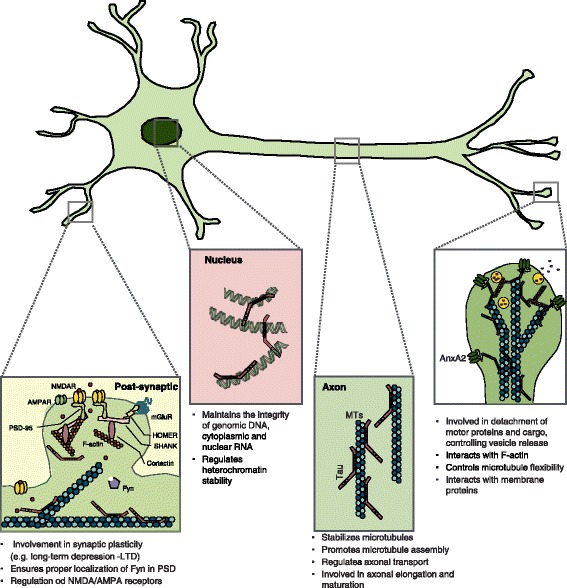
A schematic representation of the suggested role(s) of Tau in different subcellular compartments such as neuronal axon, nucleus, post- and pre-synaptic compartments

Tau protein was discovered in 1975 [1] and its original name was given by Marc Kirschner as a “factor” that was “associated” with tubulin promoting its self-assembly into microtubules (MTs). Indeed, Tau was one of the first microtubule-associated proteins (MAPs) to be characterized. Its discovery [2–7] was followed by the characterization of Tau as an axonal protein in neurons [7, 8]. In living cells, the bulk of Tau protein is attached to microtubules and stabilizes them; hence its role in the microtubule-based cytoskeleton was accepted as the standard Tau function (see also Fig. 1). Note that a non-standard role for Tau in relation to RNA, DNA, or actin binding was suggested almost four decades ago [9–11] (for review see [12, 13]), but did not maintain its impetus [14].

A major new line of Tau research was established after the discovery that Tau is a major component of abnormal protein deposits in the brains of patients suffering from AD, a neurodegenerative disorder presenting brain atrophy and memory loss. Indeed, Tau was the first protein to be identified as the main component of neurofibrillary tangles (NFTs), one of the main histopathological hallmarks of AD [15–19]. In the early 1980’s, amyloid beta (Aβ) was also found to be deposited in extracellular amyloid plaques [20] based on results obtained with Down syndrome brains [21] and these amyloid plaques accepted as the second histopathological characteristic of AD brains. During the 80’s, different pathological Tau modifications such as aberrant hyperphosphorylation, conformation, ubiquitylation, acetylation, truncation and aggregation and others, were also identified in AD brains and other neurodegenerative disorders [18, 22–26], now collectively called Tauopathies. The term Tauopathy was used for the first time to define the family with the +3 MAPT mutation [27] (see also the article “*What is the evidence that the spread of tau pathology occurs via a prion-like mechanism?”* in this issue). In addition, increasing research efforts have been focused on elucidating the physiological versus pathological properties of Tau, investigating mechanisms of neuronal dysfunction and pathology attributed to loss-of-normal function or gain-of-toxic Tau properties in AD and other neuronal pathologies with diverse etiologies e.g. epilepsy, excitotoxicity, and environmental stress [28–30].

### Transcriptomic and proteomic profile of tau – What do we miss?

Tau protein in humans is encoded by the *MAPT* gene, which is located on chromosome 17q21 and comprises 16 exons, where exons 1(E1), E4, E5, E7, E9, E11, E12 and E13 are constitutive, and the others are subjected to alternative splicing. E0 and E1 encode for 5′ untranslated *MAPT* mRNA sequences, where E0 as part of the promoter, is transcribed but not translated [31, 32]. Alternative mRNA splicing of exons E2, E3 and E10, generates 6 isoforms in the adult human brain. These isoforms differ with regard to the number of 29 residue-long near-amino-terminal inserts, encoded by E2 and E3. Isoforms containing 0, 1 or 2 inserts are known as 0 N, 1 N or 2 N, respectively. Isoforms can also be categorized depending on whether they contain 3 or 4 near carboxyl-terminal repeats (3R and 4R, respectively). The second repeat (R2) is encoded by the alternatively spliced E10, whose inclusion yields the 4R isoform, but it is excluded in mRNA encoding, 3R–Tau [33, 34].

Expression of the six Tau isoforms is developmentally regulated [35], with the smallest and most highly phosphorylated 0N3R (352 a.a) being most abundant in fetal (human or rodent) brains. The Tau expression pattern is modified post-developmentally with a reduction in 0N3R levels and the presence of all six Tau isoforms in the adult human with the levels of 3R and 4R isoforms roughly equal and underrepresentation of the 2 N species [35]. In contrast, there are mainly 4R isoforms in the adult rodent brain [36, 37]. It is unclear at the moment whether such apparent differential regulation of isoform expression of their respective Tau ortholog occurs in invertebrates such as Drosophila or non-mammalian vertebrates [38]. The role of the axon initial segment in the axodendritic sorting of different Tau isoforms has been recently reported in rat cortical neurons [39]. However, these observations raise mostly unanswered questions on whether atypical Tau functions involve particular isoforms exclusively or preferentially. Moreover, the potentially differential distribution of Tau isoforms in the brain and/or their intraneuronal-specific localization remains mostly unanswered.

The round table discussion explored the evidence ascribing atypical Tau functions and debated whether establishment and understanding of these functions would be better unraveled by thorough identification of the intracellular and brain region-specific localization of the different isoforms, or whether its localization alone, disregarding the isoform complexity, can yield expedient understanding of its function(s) in the different locations. The complex nature of the isoform-specific approach in relation to the mouse, rat, human and fruit fly brain was debated. Evidence arguing that a fruitful approach does not necessitate knowledge of isoform-specific subcellular localization was presented from Amrit Mudher suggesting that human Tau isoforms in the Drosophila model present differential phenotypes consistent with unique isoform-specific pathophysiological functions [40]. Consistent with this view, recently published work by Bart Dermaut described a pathological role for the 4R, but not the 3R, Tau during Drosophila development [41], a further demonstration of the utility of this model in addressing such questions in vivo.

A significant point raised in the discussion was the apparent lack of a map detailing Tau isoform-specific or differential localization in a vertebrate brain. However, some published evidence and unpublished work from Maria Spillantini’s lab indicates Tau isoform-specific distribution in the brain, in support of previous studies suggesting considerable regional variation in Tau expression [34]. Hence, Tau mRNA and protein levels in the neocortex are 2~fold higher than those in the white matter and cerebellum [42]. Moreover, splicing of the *MAPT* primary transcript also presents regional differences. For example, 0N3R Tau is lower in the adult cerebellum than in other regions [42, 43]. Recent findings from Jürgen Götz’s Lab demonstrated that the 1 N tau isoform is highly expressed in the murine pituitary gland, compared to the cortex or hippocampus, but is weaker in the olfactory bulb. The 2 N isoform is enriched in the cerebellum but its levels are also reduced in the olfactory bulb. In contrast, the 0 N isoform presents the highest expression in the olfactory bulb followed by the cortex [44]. These variations may contribute to the well-known differential vulnerability of the distinct brain regions to Tau pathology, while specific disturbances of the normally 1:1 4R/3R ratio are associated with distinct Tauopathies [45, 46]. The regions in which 3R is more abundant could also be associated with higher proliferation or stem cell presence such as the dentate gyrus and olfactory bulb [47].

In terms of intracellular localization, based on immunocytochemical staining, Tau is mainly found in the axons of mature neurons (see Fig. 1). However, it is ubiquitous in immature neurons distributing apparently equally in the cell body and neurites, but becomes primarily axonal during neuronal maturation and emergence of neuronal polarization. This intracellular sorting of Tau is accompanied by a shift towards the higher-molecular-weight 4R isoforms and reduced phosphorylation [4, 48–50]. Furthermore, the axonal presence of Tau differs between the ends of the axon, as it is mostly associated with MTs at the distal end of the axon close to the growth cone [51, 52] (see Fig. 1). However, Tau intraneuronal distribution in the human brain is still under debate as nearly equal amounts of Tau were described in the human cerebral gray (somatodendrites) as the underlying white matter (axons) using biochemical assays [53].

Tau phosphorylation is suggested to be involved in this intra-axonal sorting since it was also found to vary along the length of the growing axon. A phosphorylation gradient is evident, with a gradual change from phosphorylated to dephosphorylated Tau going from the soma towards the growth cone [54]. As MTs are more dynamic in the distal regions of growing axons, and dephosphorylation at certain sites increases its affinity towards MTs, these findings suggest that Tau in the growing axon has additional functions to increasing MTs stability. Indeed, a novel function for Tau as a regulator of End Binding proteins 1 and 3 (EB1/3) in extending neurites and axons of developing neurons was presented and discussed by C.L. Sayas [55]. EBs are the core plus-end tracking proteins (+TIPs), which accumulate at the growing ends of MTs, regulating their dynamic state. The current evidence suggests that the interaction between Tau and EBs is direct and dependent on Tau phosphorylation [56] and is dramatically increased by NAP, a neuroprotective peptide, derived from activity-dependent neuroprotective protein [57]. These recent findings offer new insights on the interaction of Tau with other cytoskeletal proteins (e.g. EBs) in mature neurons while future studies should further monitor the role of Tau-EB interaction under pathological conditions e.g. Alzheimer’s disease and other Tauopathies [58].

Multiple studies have provided evidence of low levels of Tau localizing in different intracellular compartments such as the nucleus, nucleolus, plasma membrane, dendrites and dendritic spines (see Fig. 1), as well as in association with various cellular organelles such as the ribosomes, endoplasmic reticulum and the Golgi apparatus [13]. The mechanisms driving this apparent intraneuronal Tau sorting are still not well understood, but evidence suggests that it could occur both at the mRNA or protein level. One of the suggested mechanisms for Tau sorting is based on selective Tau transport into axons or selective degradation in dendrites [59]. An alternative hypothesis suggests that somehow Tau possesses a higher affinity for axons than dendrites [59], consistent with its observed elevation in the axonal compartment. In line with this notion, evidence from Li and colleagues indicated that the axon initial segment (AIS) operates as a barrier against retrograde diffusion of Tau into the dendrites and that Tau phosphorylation and its interaction with MTs is essential for this barrier to be maintained [60]. It has been reported that Tau acetylation destabilizes the AIS cytoskeleton and promote the somatodendritic mislocalization of Tau [61].

Furthermore, the projection domain of Tau interacts with membrane complexes and cytoplasmatic components [62], suggesting that it is a differential property of the higher molecular weight isoforms (1 N and 2 N) that possess these domains. It is proposed that Tau interaction with annexin A2, through domains outside those binding MTs [63], contributes to its axon specific distribution and this interaction is modulated by phosphorylation [64], Indeed, Tau mutations leading to aberrant interaction with annexin A2 are likely responsible for the redistribution of Tau away from the axons to the somatodendritic compartment [63].

Interestingly, the intracellular sorting of Tau in different compartments seems to be isoform-dependent [44]. For instance, it has been reported that 1 N isoforms are localized mainly to the nucleus, 0 N isoforms primarily to the cell bodies and axons whereas the 2 N isoforms are elevated in axons and cell bodies [44]. Indeed, Marie Galas and colleagues have recently shown that overexpression of the 0N4R Tau isoform in Tau-knock out (Tau-KO) mouse neurons led to its cytoplasmic localization. Moreover, this Tau isoform goes mostly to the nucleus when tagged with a Nuclear Localization Signal (NLS) [65]. However, such compartment-specific Tau isoform mapping has not been performed in the human brain.

The complexity of using the isoform-specific approach to define other Tau functions was also pointed out, further elaborated because of the existence of Tau species in addition to the six main isoforms [66, 67]. In fact, alternative splicing could yield up to 30 different potential Tau isoforms [32, 66]. In addition, Tau can also be localized in peripheral nervous system (PNS) neurons which express a district high molecular weight (HMW) Tau species [68–70]- see also below. This is further complicated by the fact that different Tau transcripts have been described in the literature, including a 2 kb transcript in human cells, that utilize alternate polyadenylation sites on the Tau pre-mRNA, albeit of unknown significance. The 2 kb transcript was found to code for a major nuclear species of Tau [71] and has also been reported in the human frontal cortex by Michel Goedert [19] and in testicular spermatid manchette [72]. The presence of Tau in the sperm and testis has also been reported independently [73, 74]. It is not clear whether the isoform-specific distribution of Tau to either the nucleus, soma and axons reported in the murine brain [44] is dictated by different transcripts (2 kb and 6 kb), or whether analogous transcripts exist in other species e.g. fruit fly. Therefore, unraveling this complexity would provide a better understanding of the isoform-specific localization and function of Tau from the transcript to protein level.

In support of several articles describing a nuclear role for Tau in RNA and DNA protection [50, 75, 76], recent findings from Marie Galas and Eliette Bonnefoy’s teams suggest a structural role in pericentromeric heterochromatin (PCH) architecture, which is impaired in AD brains and a regulatory function for Tau in the expression of PCH lncRNA [65]. Recently, a novel role of Tau in ribosomal DNA transcription and stability has been reported in cells from Bloom’s syndrome patients [77]. Consistent with these findings, data presented by the Serpell Lab provided evidence for a role of Tau in nucleolar transcriptional regulation. Furthermore, extending previous work [78], Alberto Rabano described Tau Nuclear Indentations (TNI) in the entorhinal cortex of early AD patients, which are immune-reactive only to non-phosphorylated Tau epitopes, a potential early marker, and mechanism for the disease. These TNIs may lead to loss of nuclear integrity similar to the effects of lamin invaginations that were reported in the AD brain by the Feany lab [79]. Moreover, the work presented by Bart Dermaut indicated that human Tau expression in Drosophila led to mitotic defects and aneuploidy, similar to the accumulation of aneuploidy observed in splenocytes of Tau-KO mice [80]. This suggests yet another role for Tau in chromosome stability, in agreement with previous studies utilizing peripheral cells from Tauopathy patients [81].

Collectively, the differential distribution of Tau and its isoforms in various cell compartments may reflect distinct subcellularly compartmentalized roles; if so, then disturbances in this Tau sorting and compartmentalization could trigger neuronal dysfunction and neurodegeneration as discussed below. As suggested by different round table participants, future studies should explicitly state the Tau isoform employed in their models, as well as monitor its sub-cellular localization, such that findings can be interpreted taking into consideration that they may not pertain to all Tau isoforms.

### Tau splicing and isoform expression in neuronal function and malfunction

Splicing of the *MAPT* primary transcripts is tightly regulated by several different mechanisms, while its dysregulation and the resulting imbalance of 4R/3R Tau protein and transcripts is causally related to Tau pathology (for review see [24, 82]). The RNA-binding protein Fused in Sarcoma (FUS) may promote skipping of E3 and E10, as FUS knockdown has been reported to increase the expression of 2 N and 4R Tau isoforms [83]. Recently, knockdown of FUS and of Splicing Factor, Proline and Glutamine-rich (SFPQ) was shown to affect E10-related splicing leading to increased 4R/3R ratio, hyperphosphorylation, and neurodegeneration [84]. Small non-coding RNAs (miRNAs) can also influence Tau splicing. For example, miR-132 reduces 4R expression in mouse neuroblastoma cells [85], and miR219 represses Tau protein synthesis by binding to the 3′ untranslated region of the mRNA [86, 87]. Another mechanism that could be linked to the regulation of Tau isoform expression is the formation of ribonucleoprotein granules that results in a shift towards the expression of larger Tau isoforms (see below).

New evidence supports a bi-directional interaction between Tau and the cellular transcriptome. For example, Tau itself can bind to tRNA, a property that may favor Tau fibril formation [88, 89]. Consistent with its role in regulating the cellular transcriptome, unpublished work from Bruno Lefebvre in Luc Buée’s lab provided evidence for an interaction of Tau with the DEAD-box RNA helicase DDX5, supporting a novel role in RNA metabolism and surveillance. Moreover, accumulating evidence from various labs supports a profoundly important role for RNA binding proteins (RBPs) in Tau biology. All RNA is trafficked throughout the neuron in granules composed of RBPs and mRNA. These RBPs appear to spontaneously coalescence into a state resembling lipid droplets or vesicles [90] allowing the RBP/RNA complexes to form granules, which could be considered membraneless organelles. The Tau mRNA-binding proteins RAS GTPase-activating protein-binding protein 1 (G3BP1) and the minor histocompatibility antigen H13 or IMP1 for example, promote the formation of such granules. This leads to a shift towards the production of larger Tau isoforms and therefore, controls axonal sprouting [91] among other functional changes.

Accordingly, a recent study by Akihiko Takashima’s team demonstrated co-localization of Tau mRNA with two RNA binding proteins (RBPs), Stau1 and FMRP, which function as transport proteins. Interestingly, glutamate-driven neuronal activity stimulates local translation of Tau mRNA within mRNP granules in the somatodendritic compartment where the protein accumulates and becomes hyperphosphorylated [92]. Furthermore, another type of RBP/RNA complexes, the Stress Granules (SGs), was recently shown to contribute to Tau pathology and neurodegeneration. SGs normally sequester non-essential mRNA during stressful conditions, allowing the cell to direct protein synthesis towards cytoprotective proteins [93, 94]. However, persistent SG formation seems to be pathological as it directly stimulates Tau aggregation as shown by different studies from the Benjamin Wolozin’s lab [93, 95]. Moreover, Tau was also shown to stimulate the formation of SGs indicating that its interaction with the mRNA trafficking machinery maybe bi-directional [95]. On the other hand, alteration of cytoplasmic eIF2α and reduced SGs formation has been recently reported in the THY-Tau22 tauopathy mouse model under acute hyperthermic stress, raising further questions about the interplay of Tau protein and the cellular transcriptome under physiological and pathological conditions [96].

### Novel aspects of physiological functions of tau

Tau hyperphosphorylation and aggregation are well-established key events in AD neuropathology [22]. Although the impact of these disease-associated changes on Tau’s microtubule binding function has been reported [97–101], its effect(s) on atypical Tau functions are not yet known. Thus, the overall contribution of such disease-associated changes to the potential loss or alteration of novel Tau function(s) and AD pathology is still unclear.

Recent experimental evidence from different teams suggests that Tau loss impacts on neuronal function in the CNS and PNS impinging upon different behavioral domains. While deletion of Tau does not precipitate gross behavioral or neurostructural alterations in young/adult mice [28, 102–104], previous work has shown that loss of Tau impacts on mechanisms of synaptic plasticity, as Tau-KO animals exhibit deficits in hippocampal LTD [105] and LTP [106]. Moreover, these synaptic changes may be aggravated by aging, as 20-month-old Tau-KO animals also exhibit reduced excitatory synaptic markers and reduced active forms of other MAPs, implicating the cumulative loss of functional MAPs and acetylated tubulin in synaptic deficits and cognitive impairment triggered by aging and loss of Tau [102].

Another age-related phenotype that has been described recently is related to a novel role of Tau in regulated brain insulin signaling [107]. This recent study by David Blum and Luc Buée showed that Tau deletion leads to an impaired hippocampal response to insulin. This could explain the spatial memory deficit upon Tau deletion and peripheral glucose metabolism impairments associated with hypothalamic insulin resistance. In line with this animal evidence, human genetic analyses link the Tau haplotype to glucose homeostasis. The regulatory role of Tau in insulin signaling involves two different nodes. First, Tau-KO mice exhibit higher phosphorylation of IRS-1 at the inhibitory S636 site, known to be linked to insulin resistance in the AD and Tauopathy brain [108, 109], and possibly involve downstream kinase activation. Second, Marininak’s study demonstrates that Tau levels tend to reduce the ability of PTEN lipidphosphatase to dephosphorylate PIP3 into PIP2, an important step in downstream insulin signaling. These findings raise the hypothesis that pathophysiological Tau loss-of-function favors brain insulin resistance, which is likely instrumental for the cognitive and metabolic impairments described in AD patients [107].

Furthermore, Tau involvement in myelination through its interaction with the kinase Fyn and MTs has been also described [110–112]. Accordingly, ultrastructural and biochemical analysis of Tau-KO animals demonstrated a hypomyelination phenotype in sciatic nerves of young and adult Tau-KO mice [113] originating in small caliber axons that also exhibit microtubule alterations [114] and altered pain processing [113]. Moreover, these Tau-dependent morphofunctional effects exhibited an age-progressive phenotype with old Tau-KO animals presenting degenerating myelinated fibers and progressive hypomyelination of large-diameter, motor-related axons accompanied by motor deficits [115]. Other studies have also related the age-dependent motor deficits of Tau-KO animals with an age-related loss of substantia nigra (SN) dopaminergic neurons [116] (but also see ref. [103]). Interestingly, similar motor deficits, such as reduced motor strength and coordination, were also found in old animals lacking 4R–Tau, suggesting a potential role for this large isoform in age-dependent development of motor deficits [117]. Note that, although Tau is expressed in both CNS and PNS, the isoforms expressed in adult CNS differ from the HMW Tau isoforms (“big Tau”) found mainly in PNS (e.g., sciatic nerves) but also in optical nerves and retina [70, 118–120]. Expression of HMW Tau isoforms may confer increased stabilization and spacing of MTs [121, 122] but to date, our knowledge about Tau function in the PNS is very limited.

### Tau protein as key regulator of brain neuroplasticity and neuropathology

In contrast to axons, a small amount of Tau is present in dendrites and dendritic spines under normal, physiological conditions but its function therein has not been well characterized [123, 124]. It is suggested that in this compartment, Tau may regulate synaptic plasticity as pharmacological synaptic activation induces translocation of endogenous Tau from the dendritic shaft to excitatory post-synaptic compartments in cultured mouse neurons and in acute hippocampal slices [125]. Through its interaction with several cellular partners such as tubulin, F-actin, Src family kinases, Tau may play an important role in mediating alterations in the cytoskeletal structure of dendrites and spines as well as synaptic scaffold and signaling [126]. This notion is further supported by the fact that mechanisms of synaptic plasticity are impaired in Tau-KO animals [105, 106] while Tau phosphorylation in specific epitopes is suggested to be critical for synaptic plasticity [127].

Localization of Tau at the synapse has been the focus of several recent reports aiming to determine whether and why Tau is located at the pre-synaptic, the postsynaptic, or both compartments [124]. We now know that Tau interacts directly with filamentous (F) actin [128], localized both in presynaptic boutons and in the head and neck of dendritic spines [129]. Furthermore, using synaptosomes derived from healthy and AD brains, recent studies demonstrated that Tau is present in both pre- and post-synaptic compartments [124], although phosphorylated Tau was found in greater amounts in the postsynaptic sites. Furthermore, using a mouse Tauopathy model expressing the FTDP-17 associated mutation P301L, PHF–Tau was found in both pre- and post-synaptic compartments suggesting that Tau distribution changes in the context of disease [130].

There are several potential mechanisms by which Tau could affect synaptic function and neuronal excitability. It may directly influence synaptic function since, as described above, Tau has been shown to be localized within both pre- and post-synaptic compartments, possibly due to its interaction with other essential synaptic proteins. Further analysis has shown that the phosphorylation status of Tau is modulated through NMDA receptor activation [123]. However, unphosphorylated species are also present in this compartment, suggesting that in synapses, Tau is likely to oscillate between phosphorylated and non-phosphorylated states [123]. Very recently, Kobayachi and colleagues provided evidence that physiological neuronal activity stimulates local translation and phosphorylation of Tau [92]. These data strongly suggest that in dendritic compartments, Tau is involved in physiological synaptic function. However, dendritic localization is more extensively studied in the context of AD pathology, where phosphorylated Tau is missorted into dendrites but also into dendritic spines, causing synaptic dysfunction by suppressing AMPA receptor-mediated synaptic responses, through disruption of post-synaptic targeting and anchoring of glutamate receptors [131].

At the synapse, Tau has been shown to associate with the PSD complex [132], and function in targeting Fyn, a Tyrosine Kinase that belongs to the Src family, to postsynaptic compartments and to be involved in coupling NMDARs to PSD95 [110, 133, 134]. The interaction of Tau with Fyn appears to be essential for targeting Fyn to PSD, where it regulates NMDA receptor function through phosphorylation [135] and the interaction of Fyn with membrane-associated proteins of the plasma membrane [136, 137]. The interaction with Fyn is regulated by the phosphorylation status of Tau, and therefore can be disrupted in disease, when its phosphorylation pattern is altered [133, 136, 138] (see also Fig. 1).

Cumulative evidence from experimental studies using genetic attenuation of Tau levels suggests that it mediates, at least in part, the detrimental effects of Aβ on neuronal function. In fact, Tau ablation has been shown to protect against Aβ-driven AD brain pathology, neurotoxicity and memory impairment [139–142]. One of the possible mechanisms through which Tau could trigger neuronal and/or synaptic malfunction is based on its Aβ-driven missorting at dendritic spines, a potential early event in AD, preceding the manifestation of detectable neurodegeneration [131, 143]. Recent evidence demonstrated that the intracellular distribution of Tau depends critically on the phosphorylation status of the protein [144]. Accordingly, hyperphosphorylation seems to be necessary for Tau missorting at synapses as mimicking hyperphosphorylation by pseudophosphorylation, mislocalizes it to dendritic spines, an effect not observed with phosphorylation-deficient protein [131]. Importantly, Aβ is a well-known trigger of Tau missorting and dendritic collapse [110, 123, 131, 145–147], leading to increased postsynaptic targeting of Fyn [110]. Fyn selectively modulates the function of GluN2B-containing NMDARs, by phosphorylation of the GluN2B on the Y1472 epitope [110, 148]. This phosphorylation is known to stabilize GluN2B at the postsynaptic density linking NMDARs to downstream excitotoxic signaling due to their overexcitation [110, 148].

Recent results from Dr. Sotiropoulos’ team extended the contribution of Tau hyperphosphorylation and missorting to the detrimental effects of exposure to lifetime stress. Stress-dependent Tau missorting may precipitate the dendritic and synaptic malfunctions implicated in the development of neuropsychiatric pathologies such as depression, a known risk factor for AD. These studies demonstrate that chronic stress causes dendritic atrophy, reduced neurogenesis and synaptic deficits in hippocampal integrity leading to cognitive and mood deficits in a Tau-dependent manner [28, 104, 149, 150]. Chronic stress triggers Tau hyperphosphorylation and synaptic missorting of Tau, increased postsynaptic targeting of Fyn and elevation of pGluN2B at the postsynaptic density representing a potential mechanism of stress-driven neurotoxicity. Importantly, all these changes could be abrogated by the ablation of Tau in Tau-KO animals. This, in turn, reveals the protective role of Tau reduction against the establishment of stress-driven hippocampal pathology. This observation is in line with other approaches using Tau-downregulation strategies to tackle neuropathologies with diverse etiology such as AD, epilepsy, Dravet syndrome, excitotoxicity, stress-driven depression [29, 110, 140, 151].

Collectively, these studies highlight Tau protein as a key regulator of neuronal plasticity and pathology in and beyond AD. Indeed, previous studies have shown that Tau hyperphosphorylation and neuronal/synaptic atrophy is also triggered by different intrinsic and extrinsic conditions such as acute stress [152], hypothermia [153], hypometabolism [154], and hibernation [155] in a reversible manner. Thus, future studies are necessary to identify the potential threshold/“point of no return” between Tau-related neuroplasticity and neuropathology during brain aging that may contribute to our understanding of the various precipitating factors of AD as well as of a broader spectrum of brain pathologies.

## Future directions

This review further emphasizes the view of Tau as a multifunctional protein. However, it is evident that our knowledge about its atypical/non-standard functions is very limited and could represent only the tip of the Tau “iceberg”. Thus, a main goal of the field is to clarify the exact molecular mechanisms underlying the already-described Tau functions as well as decipher novel Tau physiological roles and their potential involvement in neuropathology. Many participants of this round table discussion suggested that future research efforts should focus on the detailed monitoring of Tau interacting partners, different subcellular locations and post-translational modifications of Tau, as well as the potential implication of various pools of Tau isoforms, aiming to understand their role on Tau action(s) and its role in neuronal (mal)function. Another important issue will be to define the functions of extracellular Tau (see also the article “*What is the evidence that the spread of tau pathology occurs via a prion-like mechanism?”* in this issue) and their role in the pathophysiological processes.

## Conclusions

Although Tau protein was found more than 40 years ago, our knowledge about its role(s) in brain function/malfunction is mainly based on its involvement in AD pathology and other Tauopathies. While we are aware that this review may not cover the entire field (e.g. extracellular Tau –see also above), this short report aimed to summarize recent findings that were presented and discussed in 1st EuroTau meeting related to novel and atypical roles of Tau adding unique insights to our limited knowledge on Tau-related neuronal (mal)function. In light of the accumulating evidence supporting the potential involvement of Tau in neuronal pathologies with diverse etiology, the findings presented and discussed here may trigger novel lines of research that will contribute to better understanding of Tau biology and identify potential therapeutic targets against brain aging and pathology.

## Abbreviations

+TIPs: core plus end tracking proteins
AD: Alzheimer’s Disease
AIS: Axonal Initial segment
AMPA: α-amino-3-hydroxy-5-methyl-4-isoxazolepropionic acid
Aβ: amyloid-β
CNS: Central nervous system
DDX5: DEAD-box RNA helicase 5
DNA: Deoxyribonucleic acid
EBs: End binding proteins
eIF2a: Eukaryotic translation initiation factor 2A
FMRP: fragile X mental retardation protein
FTDP-17: Frontotemporal dementia with parkinsonism linked to chromosome 17
FUS: RNA-binding protein fused sarcoma
G3BP1: GTPase-activating protein-bindingprotein 1
GluN: Glutamate [NMDA] receptor subunit
H13: Minor histocompatibility antigen
HMW: High molecular weight
IMP: Insulin-like growth factor-II mRNA-binding proteins
IRS-1: Insulin receptor substrate 1
Kb: Kilo base
KO: Knockout
lncRNA: Long non-coding RNA
LTD: Long-term depression
LTP: Long-term Potentiation
MAPs: Microtubule associated proteins
MAPT: Microtubule Associated Protein Tau
miRNA: micro RNA.
mRNA: messenger RNA.
MTs: Microtubules.
NAP: Nucleossome assembly protein.
NFTs: Neurofibrillary Tangle.
NLS: Nuclear Localization Signal.
NMD: Nonsense-mediated mRNA decay.
NMDA: N-methyl-D-aspartate.
PCH: Pericentromeric heterochromatin.
PHF: Paired-helical filaments.
PIP2: Phosphatidylinositol biphosphate.
PIP3: Phosphatidylinositol triphosphate.
PNS: Peripheral Nervous System.
PSD: Post-synaptic Density.
PTEN: Phosphatase and tensin homolog.
RBPs: RNA binding protein.
RNA: Ribonucleic acid.
SGs: Stress Granules.
SN: Substantia Nigra.
TNI: Tau Nuclear Indentations.
